# The diurnal course of salivary cortisol and alpha-amylase on workdays and leisure days in teachers and the role of social isolation and neuroticism

**DOI:** 10.1371/journal.pone.0286475

**Published:** 2023-05-31

**Authors:** Sandra Schneider, Martin grosse Holtforth, Alexander Wettstein, Gabriel Jenni, Fabienne Kühne, Wolfgang Tschacher, Roberto La Marca

**Affiliations:** 1 Clinical Psychology and Psychotherapy, Department of Psychology, University of Bern, Bern, Switzerland; 2 Department of Research and Development, University of Teacher Education Bern, Bern, Switzerland; 3 Psychosomatic Medicine, Department of Neurology, Inselspital, Bern University Hospital, University of Bern, Bern, Switzerland; 4 Experimental Psychology Division, University Hospital of Psychiatry and Psychotherapy, University of Bern, Bern, Switzerland; 5 Clinica Holistica Engiadina, Centre for Stress-Related Disorders, Susch, Switzerland; 6 Clinical Psychology and Psychotherapy, Department of Psychology, University of Zurich, Zurich, Switzerland; The John Paul II Catholic University of Lublin, POLAND

## Abstract

Teachers are among the occupational groups with the highest sick leave rates due to workplace stress and burnout symptoms. A substantial body of research has suggested social isolation and neuroticism to be related to physiological stress activity. However, the relationship between such characteristics and stress experiences has rarely been studied in conjunction with physiological stress indicators in the teachers’ natural settings. Thus, the present study examines salivary cortisol and α-amylase as physiological stress indicators on teachers’ work and leisure days and their relationship with social isolation. Furthermore, we test whether neuroticism moderates the relationship between social isolation and salivary biomarkers. Forty-two teachers completed questionnaires assessing social isolation (Trier Inventory for the Assessment of Chronic Stress) and neuroticism (Big-Five Inventory). Participants collected eight saliva samples on three days, two workdays, and one leisure day to measure the concentration of cortisol and α-amylase as biomarkers of the hypothalamic-pituitary-adrenal (HPA) axis and the autonomic nervous system (ANS), respectively. Results showed a significantly higher Cortisol Awakening Response (CAR) and diurnal cortisol slope (DCS) on workdays than on the leisure day but no significant differences regarding measures of α-amylase. We found a significant positive relationship between social isolation and the CAR on the leisure day but no association with the α-amylase measures. Furthermore, after controlling for confounders, social isolation was unrelated to neuroticism, and the latter did not moderate between social isolation and the CAR. Our findings suggest an association between social isolation and the HPA axis, i.e., the CAR, but do not support an association with the ANS, which would be indicated by the α-amylase assessments. Finally, our findings could not support an association of neuroticism with the HPA axis and ANS.

## Introduction

Teachers are among the occupational groups with the highest sick leave rates due to workplace stress and burnout symptoms [1, 2]. Exploring the relationship between teacher characteristics and stress-related indicators may increase our understanding of the factors contributing to individual stress experiences and psychosomatic complaints among teachers.

Social isolation, a multidimensional concept describing the absence or lack of social relationships and interactions with family members, friends, or peers [3], precipitates hypervigilance to social threats in the environment, such as rejection, exclusion, being evaluated negatively, and feeling unsafe [4, 5]. This may cause attentional and memory biases, making socially isolated people more likely to remember negative social events, perceive and interpret their social world as threatening, and have negative social expectations. Social interactions (e.g., in the classroom) are an essential part of the teaching profession, and the fear of being negatively evaluated and experiencing a lack of sympathy are factors identified to contribute to teacher anxiety and, eventually, burnout [6].

In addition, studies have shown that personality is a crucial determinant of how individuals appraise, cope, and react to stressors [7, 8]. Individuals high in neuroticism are more prone to avoidant coping styles such as disengagement, especially withdrawal and wishful thinking [9]. The inability of affected teachers to withdraw from potential psychosocial stress while teaching may contribute to increased stress during teachers’ workdays. Furthermore, teachers with high scores on the Big Five [10] personality trait neuroticism were found to perceive more negative emotions such as anxiety or sadness, self-doubt, self-criticism, and work-related rumination, putting them at a higher risk for burnout [11]. This leads to the assumption that neuroticism in socially isolated teachers reinforces the interpretation of social interactions as more negative and threatening, potentially contributing to heightened physiological stress activity in teachers’ daily lives.

Previous research suggests psychosocial stress to be a trigger for the activation of the salivary biomarkers cortisol [12–14] and α-amylase [15–17]. Salivary cortisol and α-amylase are biomarkers used to reflect stress-related changes regarding the hypothalamic-pituitary-adrenal (HPA) axis [18] and noradrenergic activation of the autonomic nervous system (ANS), respectively [19]. Salivary cortisol and α-amylase follow different diurnal secretion patterns. In the first 30–45 minutes after awakening, cortisol concentrations typically increase by 50–60% (cortisol awakening response, CAR), drop rapidly in the first few hours after awakening, and decline more slowly throughout the day until reaching a low point around midnight (diurnal cortisol slope, DCS) [20]. The time of day explains most (60–70%) of the variation in cortisol concentrations during the day, yet, the shape of daily cortisol rhythms varies considerably between individuals [21]. Salivary α-amylase follows the reverse pattern as cortisol, with a decrease approximately 30 minutes after awakening (α-amylase awakening response, AAR) and a continuous increase throughout the day (diurnal α-amylase slope) [22]. HPA axis dysregulation is typically manifested by an altered CAR and an attenuated DCS [21, 23], whereas ANS dysregulation is generally characterized by an attenuated AAR, indicated by a lower decrease 30 minutes after awakening and by an increased diurnal α-amylase slope [24]. Dysregulation in these diurnal secretion patterns has been associated with stress as well as poor mental and physical health outcomes [25–27].

Social isolation has been linked to both an increased CAR [28, 29] and an attenuated DCS [30–32], while research on the association between social isolation and α-amylase has been scarce and results inconsistent [33–35]. Similarly, some previous studies measuring basal cortisol throughout the day in an ambulatory assessment design indicated a link between neuroticism and basal HPA axis activity [36, 37], while other studies have found no differences [38], and some have even found decreased plasma cortisol concentrations in individuals with high neuroticism scores [39]. Several of these studies, however, measured cortisol concentration only once or on a single day. Considering the variability of cortisol concentrations within and between different days, cortisol should not be measured only at a single time point [18]. As for α-amylase, few previous studies have examined its relationship with neuroticism. In a study by Puig-Perez and colleagues [40], neuroticism was unrelated to any α-amylase measure. In contrast to these findings, Inukai and colleagues [41] found a positive relationship between neuroticism and the AAR.

Thus far, the relationship between teacher characteristics and stress experiences has been studied mainly in conjunction with self-reported stress levels [42] rather than physiological stress indicators in the teachers’ natural settings. This biased approach potentially reduces the ecological validity and generalizability of the results, as self-reports may be influenced by various individual factors, including mood and personality traits like neuroticism [43]. Combining psychological and physiological measures is essential for better understanding the relationship between cognitive processes and physiological stress activity in teachers [44] and may provide further evidence of contributing factors to teacher stress and valuable information for appropriate prevention strategies.

Therefore, in the current study, we aim to use a combination of self-reports and salivary biomarkers in an ambulatory assessment design with the following three aims: First, we compare the awakening responses and the diurnal slopes of salivary cortisol and α-amylase on two averaged workdays and one leisure day. Second, we aim to extend previous research by linking social isolation to salivary cortisol and α-amylase concentrations. Finally, we explore the moderating role of neuroticism on the relationships between social isolation and salivary biomarkers.

## Methods

### Participants

Forty-two apparently healthy teachers (28 females, *M*_age_ = 39.66, *SD* = 11.99) were recruited via flyers and emails sent to school administrations in the canton of Bern, Switzerland. Exclusion criteria were chronic illness, medication in the past two months, living and working outside the canton of Bern, long-distance flights within the last two weeks before data collection, teaching less than sixteen lessons per week (corresponding to an employment level of at least 60 percent), drug use or increased alcohol consumption (i.e., drinking more than 2 standard drinks per day), smoking over ten cigarettes per day, and pregnancy. Participants worked at 39 different schools and reported a mean of 13.35 years of teaching experience (*SD* = 11.07, range = 1–40). Participants had completed standard teacher training and were regular classroom teachers, not teachers for the gifted or students with special needs. The grade levels teachers taught ranged from kindergarten and elementary school (kindergarten to 6^th^ grade; *n* = 27), over secondary school (7^th^ to 9^th^ grade; *n* = 12), to high school and vocational school (10^th^ to 12^th^ grade; *n* = 3). The study was approved by the ethics committee of the canton Bern (no. 2019–00787), as well as the Internal Review Board (IRB) of the University of Bern, and was conducted in strict compliance with current data protection laws. The participating teachers signed informed written consent.

### Design and procedure

The present study is part of a larger, longitudinal study examining psychobiological stress in teachers [45]. All teachers were screened in a short interview to ensure inclusion and exclusion criteria were met. Participants completed online questionnaires on social isolation and neuroticism in December 2019 before the Covid-19 pandemic. Saliva samples were collected on two workdays and one leisure day in an ambulatory assessment design before and during the pandemic between January and November 2020. It is important to note that when data was collected, teachers were teaching in person, not online. The teachers were verbally and in writing instructed to refrain from drinking alcohol starting the evening before each data collection day. They were further instructed not to engage in strenuous physical activity on the three ambulatory assessment days.

### Measures

#### Self-reports

*Social isolation* was assessed with the Subscale *Social isolation* of the Trier Inventory for Chronic Stress (TICS) [46]. Participants rated the six items with a value between 1 = *never* and 5 = *very often*, depending on how often the statements applied to them during the last three months. Sample items were *"Times when I am alone too much*,*" "Times when I lack friends to do things with*,*" and "Times when I don’t have the opportunity to talk to others*.*"* This resulted in a total sum score ranging from 6 to 30 points. Cronbach’s alpha was α = .83.

*Neuroticism* was assessed using two items of the short version of the Big Five personality trait scale (BFI-10) [47] and two additional items of the NEO-FFI [48] that had shown the highest factor loadings in the norm sample. Items were *"I get depressed quickly*, *dejected"* (NEO-FFI), *"I worry a lot"* (NEO-FFI), *"I see myself as someone who is relaxed*, *handles stress well"* (reversed item, BFI-10), and *"I see myself as someone who gets nervous easily"* (BFI-10). The unidimensional structure of the combined items was tested using exploratory factor analysis. Both Bartlett’s test (p < .001) and the Kaiser-Meyer-Olkin measure of sampling adequacy (KMO = .675) indicated that the variables were suitable for factor analysis. Therefore, a Principal Component Analysis (PCA) with varimax rotation was performed. The obtained one-factorial solution explained 56% of the variance. Participants indicated the extent to which each statement described themselves adequately, from 1 = *not at all* to 5 = *a lot*. Consequently, mean values were calculated, summarizing the four items. Chronbach’s alpha of the four-item scale was α = .73.

#### Salivary cortisol and α-amylase

For the assessment of salivary cortisol and α-amylase, saliva was repeatedly collected on two workdays and one leisure day. On the workdays, participants were asked to collect saliva at the following time points: wake-up, + 30 min, +45 min, 8 am, 10 am, 12 am, 4 pm, and 8 pm. On the leisure days, aiming at equivalent measurement time points, participants were asked to collect saliva samples at the following time points: wake-up, + 30min, +45min, +2h, +4h, +6h, +10h, and +14h. Participants were asked to enter the time of sampling in a log booklet.

The three saliva collection days for each participant took place within one week. The order (workday or leisure day first) was randomized. However, due to rescheduling among two participants, 23 teachers first collected the leisure day samples, while 19 started with the workday sample collection. Participants were instructed verbally and in writing to gently chew cotton rolls (Sarstedt, Sevelen, Switzerland) for 2 minutes before placing them back in the provided container. After collection, participants stored the samples in their home freezer until a member of the research team picked them up and kept them in a freezer at -20°C at the University of Bern until biochemical analyses took place at the biochemical laboratory of the Psychological Department of the University of Zurich, Switzerland. Samples were thawed and centrifuged at 3000 rpm for 10 minutes. Afterward, free cortisol was analyzed using an immunoassay with time-resolved fluorescence detection [49], while the activity of α-amylase was analyzed using a kinetic colorimetric test [50].

For Cortisol and α-amylase, we calculated the awakening responses and the diurnal slopes (e.g., DCS (workdays) = (LNsCortisol(8 pm)—LNsCortisol(8 am))/ (time(8 pm)–time(8 am)), see Hoyt et al., 2016) [51]. The Area Under the Curve with respect to increase (AUCi) was used as measure of the awakening response (i.8e., the CAR and AAR), “emphasizing the changes over time” [52; page 919]. Due to the importance of accurate sampling time, significant deviations from the following time span led to the exclusion of the specific value: +20 min to +45 min (for the +30 min sample), +30 min to +60 min (for the +45 min sample), and within 1 hour from the predetermined sampling times (for the 8 am, 10 am, 12 am, 4 pm, and 8 pm sample), and the equivalent samples of the leisure day (see also [53]). This led to the exclusion of n = 1 participant concerning the CAR and AAR (on the leisure day) and n = 2 participants concerning DCS and diurnal α-amylase slope (on the leisure day). In determining the data collection days, we considered the menstrual cycle of our female participants to ensure that the three days of data collection did not fall within the follicular phase, as this could affect cortisol binding and HPA axis reactivity [18].

#### Statistical analyses

All data were analyzed using IBM SPSS Statistics (IBM SPSS Statistics, Version 26). Normal distribution was tested using the Kolmogorov-Smirnov test, and skewed variables were log-transformed using the natural logarithm (ln(x + 1)). Potential confounders, such as sex, age, and BMI, were tested for significant associations with the main variables by applying Mann-Whitney U tests or Pearson correlations. When confounders were significantly related, confounder-adjusted standardized residuals of the main variables were calculated [54]. Descriptive statistics were computed to investigate the study’s variables. Pearson correlations were conducted to examine the associations between cortisol, α-amylase, social isolation, and neuroticism.

Since no sphericity (Mauchly-test) was given, two-tailed non-parametric analyses of variance (ANOVAs) for repeated measures were computed using Friedman’s ANOVA [55] to test possible time effects. For post-hoc tests, as well as comparing physiological measures at different time points, two-tailed Wilcoxon signed-rank tests for multiple testing were performed.

The effect size of interactions was determined by calculating partial eta-square (partial eta2), with values indicating small (.01), medium (.06), or large (.14) effect sizes [56]. All analyses were two-tailed, with the significance level set at p < .05.

For exploratory purposes, moderations were tested using ordinary least square path analytic models in SPSS with the macro PROCESS version 4.0 [57]. CAR was entered as the dependent variable in the moderation analyses. Social isolation was entered as the predictor, while neuroticism was entered as the moderator. The interaction terms were built as the product of the mean-centered variables involved. The significance of the indirect effects was tested with a 95% confidence interval based on 5000 bias-corrected bootstrap samples.

## Results

### Salivary biomarkers during workdays and the leisure day

The Wilcoxon signed-rank test [58] was applied to examine whether the concentration of cortisol and α-amylase differed significantly between the leisure day and the workdays.

#### Salivary cortisol

Results revealed significantly lower CAR on the leisure day (*Mdn* = 39.81) compared to the workdays (*Mdn* = 201.24; z = -4.50, *p* < .001). The DCS was significantly lower on the leisure day (*Mdn* = -0.33) than on the workdays (*Mdn* = -0.47; z = -3.15, *p* = .002).

**Fig 1:**
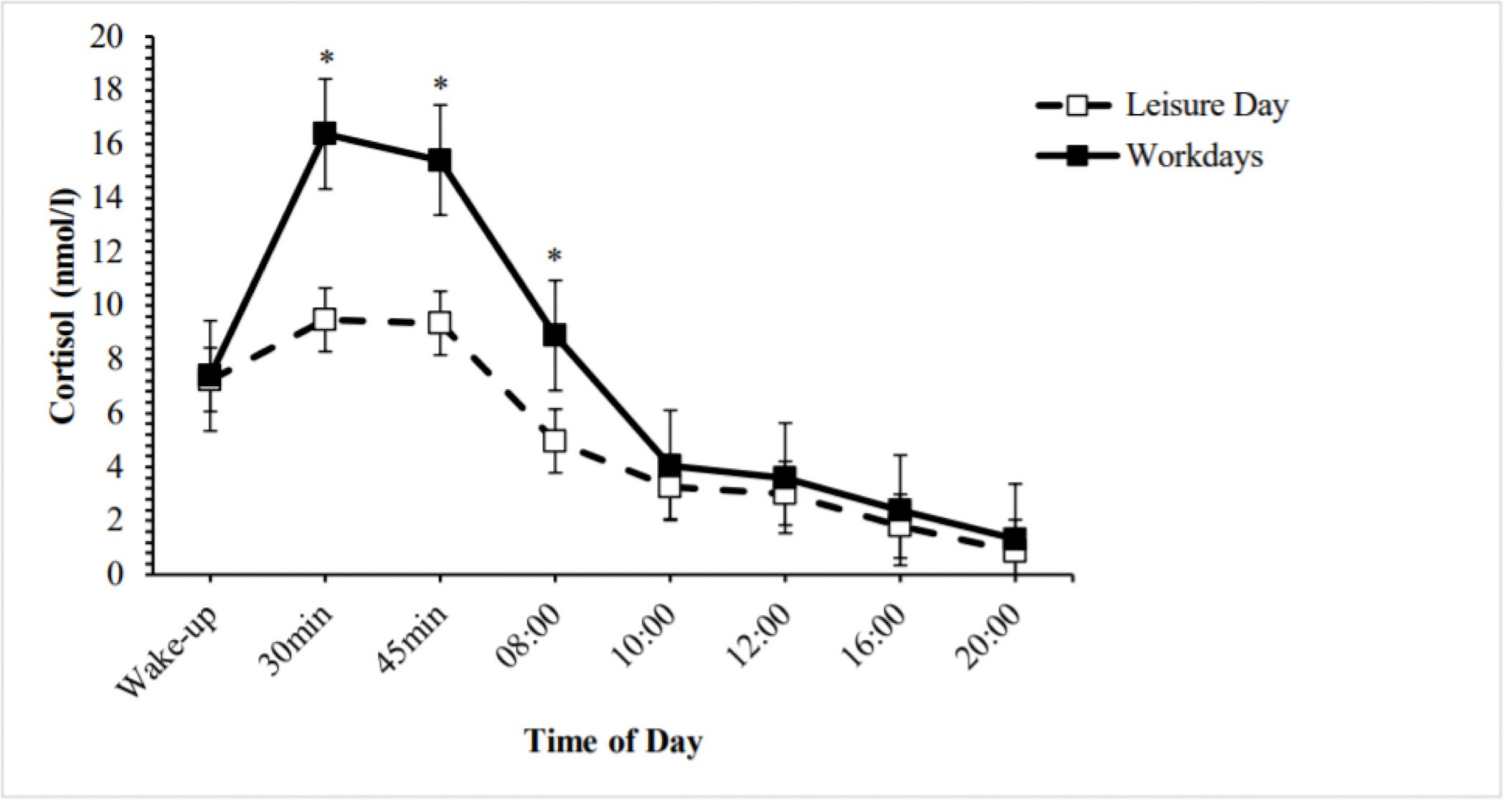
Salivary cortisol concentration during workdays (mean values of two averaged workdays; solid line) and a leisure day (dashed line). *Note*. Values represent the mean (LN) +/- standard error of the mean. Asterisks denote significant differences in cortisol concentrations between the workdays and the leisure day.

Cortisol exhibited the expected diurnal pattern on the leisure day and the workdays (Fig 1), with a peak after awakening (CAR) and a subsequent decrease [workdays: chi-square(7) = 231.04, *p* < .001; leisure day: chi-square(7) = 185.32, *p* < .001]. Cortisol concentrations 30 minutes after awakening were significantly higher on the workdays (*Mdn* = 13.86) than on the leisure day (*Mdn* = 8.89; z = -5.07, *p* < .001). The same was found at 45 minutes after awakening (*Mdn* averaged workdays = 15.14, *Mdn* leisure day = 7.47; z = -4.65, *p* < .001) and at 8 am (*Mdn* averaged workdays = 7.15, *Mdn* leisure day = 4.47; z = -3.90, *p* < .001).

#### Salivary α-amylase

For α-amylase there was no difference between the AAR on the leisure day (Mdn = -230.96) and the workdays (Mdn = -258.45, z = -0.61, *p* = .55). No difference was found between the diurnal α-amylase slope on the leisure day (*Mdn* = 1.70) and the averaged workdays (*Mdn* = 5.91; z = -0.96, *p* = .34).

**Fig 2:**
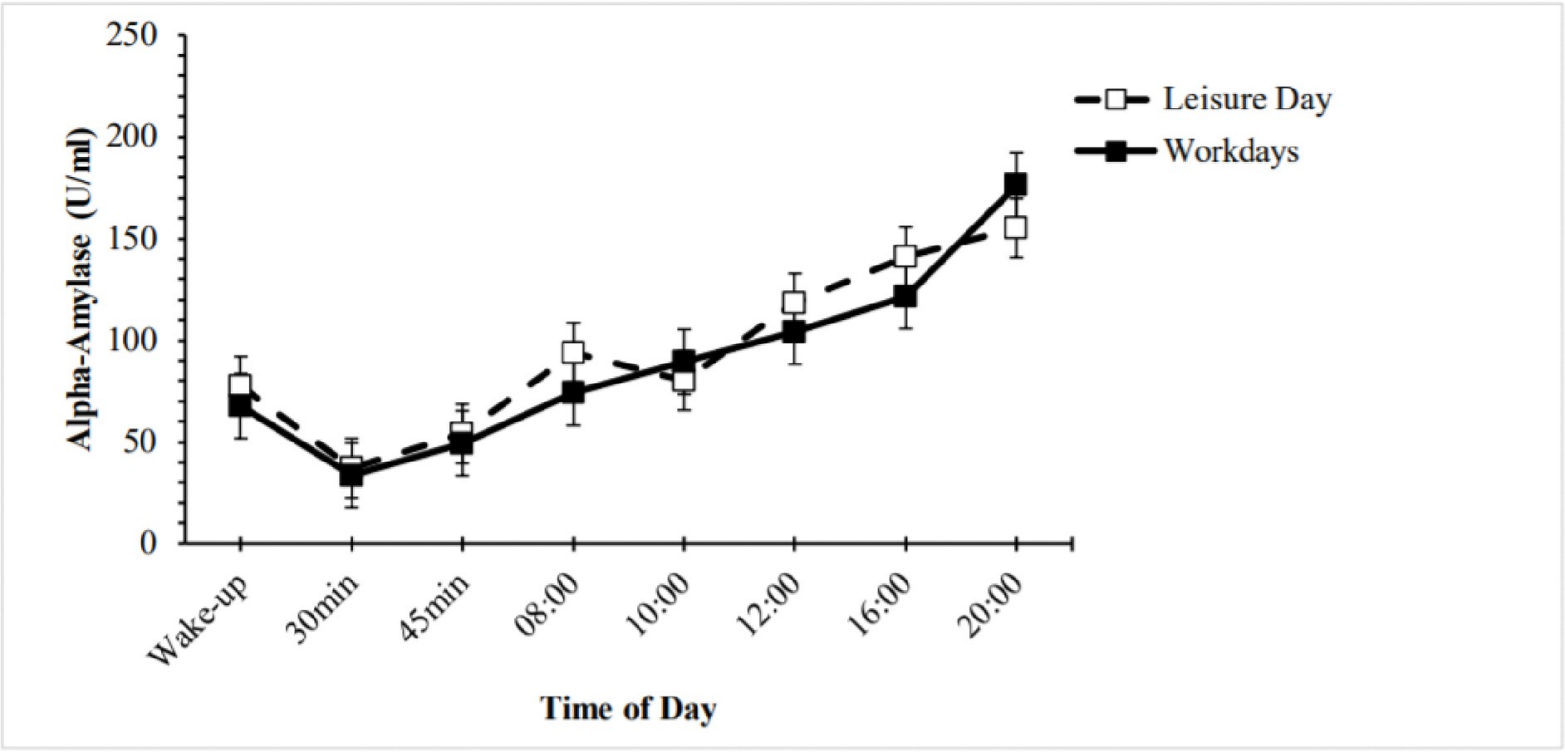
α-amylase concentration during workdays (mean values of two averaged workdays; solid line) and a leisure day (dashed line). *Note*. Values represent the mean (LN) +/- standard error of the mean.

Changes in α-amylase over time were significant on the leisure day and the workdays [workdays: chi-square(7) = 115.58, *p* < .001; leisure day: chi-square(7) = 67.76, *p* < .001]. Salivary α-amylase scores significantly decreased within the first hour of awakening and subsequently increased toward the afternoon and evening (Fig 2).

#### Intercorrelations

Table 1 shows the correlations between social isolation, salivary cortisol, α-amylase measures, neuroticism, and the potential confounders. After log-transforming the data and controlling for the confounding variables, Pearson correlations revealed that social isolation was significantly positively correlated with the CAR on the leisure day (*p* = .022) but no longer with the CAR on the two averaged workdays (*p* = .051) or with neuroticism (*p* = .389). Regarding intercorrelations with the confounders, analyses showed that BMI was significantly negatively correlated with neuroticism (*p* = .009). In other words, teachers with higher BMI had lower neuroticism scores. Age was significantly positively correlated to DCS on the leisure day (*p* = .006), indicating a flatter cortisol decrease over the day in older participants, but significantly negatively with AAR on the leisure day (*p* = .021), indicating a stronger α-amylase decrease in response to awakening in older teachers.

**Table 1:** Descriptive statistics and intercorrelations of key variables.

Variable	*M*	*SD*	1	2	3	4	5	6	7	8	9	10	11	12
**1. CAR Work**	262.60	194.50	--											
**2. CAR Leisure**	65.73	212.95	.13	--										
**3. DCS Work**	-0.63	0.53	-.17	-.16	--									
**4. DCS Leisure**	-0.36	0.24	.10	-.41*	.01	--								
**5. AAR Work**	-902.80	1973.62	-.21	-.04	.06	-.06	--							
**6. AAR Leisure**	-1119.34	3501.71	.07	-.05	.03	.09	-.04	--						
**7. DAS Work**	8.52	10.85	.05	.13	-.24	-.27	-.04	-.26	--					
**8. DAS Leisure**	5.59	14.77	-.04	.09	.31	-.01	-.14	.04	-.10	--				
**9. Social Isolation**	10.07	3.61	.31	.36*	-.04	-.17	-.07	.01	.05	.14	--			
**10. Neuroticism**	2.35	0.74	.16	.10	-.09	.09	.08	-.01	-.23	.04	.22	--		
**11. Age**	39.66	11.99	-.27	-.03	.22	.45**	.25	-.36*	-.01	.22	.06	-.11	--	
**12. BMI**	24.07	3.22	.05	-.18	.23	.06	.02	.16	.05	-.19	-.03	-.40**	.25	--
**13. Sex** ^**a**^	-	-	.02	-.01	-.13	-.02	-.03	.10	.05	-.24	.07	-.11	.35*	.12

*Note*. Biological variables as well as social isolation and age were log-transformed and confounders were considered for, where appropriate.

* *p* < .05

** *p* < .01

*N* = 42 (28 females). *Legend*: Work = Averaged workdays; Leisure = Leisure day; CAR = Cortisol Awakening Response (workdays *N* = 41, leisure day *N* = 41); DCS = Diurnal Cortisol Slope (workdays *N* = 42, free day *N* = 37); AAR = α-Amylase Awakening Response (workdays *N* = 42, leisure day *N* = 40); DAS = Diurnal α-amylase slope (workdays *N* = 42, leisure day *N* = 37); BMI = Body Mass Index.

#### Neuroticism as a moderator of the association between social isolation and CAR

In the next step, moderation analyses were conducted for significant associations between social isolation and salivary measures. First, we conducted moderation analyses with neuroticism as a moderator on the relationship between social isolation and the CAR on the workday. Before log-transforming the data and controlling for the confounding variables, the calculated moderation model was significant, *F*(3, 38) = 6.08, *p* = .002, *R*^2^ = .32, *R*^2^_adjusted_ = .27 as well as the interaction (β = -21.13, SE = 9.88, *t* = -2.14, *p* = .039). However, after log-transforming and controlling for the confounding variables, the moderation model (*F*(3, 37) = 1.59, *p* = .21, *R*^2^ = .11) no longer showed a significant interaction (β = -.06, SE = 0.15, *t* = -0.42, *p* = .68). Next, we analyzed the moderating role of neuroticism on the relationship between social isolation and CAR on the leisure day. The calculated moderation model was not significant, *F*(3, 37) = 1.86, *p* = .15, *R*^2^ = .13. The interaction was also not significant (β = -.07, SE = 0.17, *t* = -0.43, *p* = .67). Other moderation models between social isolation and biomarker measures with neuroticism as a moderator were analyzed, but no significant results were found.

## Discussion

This ambulatory-assessment study aimed 1) to compare concentrations of two salivary biomarkers (salivary cortisol and α-amylase) on two averaged workdays and a leisure day and 2) to examine the relationship between social isolation, neuroticism, and these biomarkers, and 3) to explore the moderating role of neuroticism on the relationships between social isolation and the salivary biomarkers in teachers.

Comparisons of salivary cortisol concentrations between the averaged workdays and the leisure day revealed significantly higher levels on the workdays than on the leisure day regarding the awakening responses and a significant steeper decrease during the working days. However, for salivary α-amylase, there were no significant differences between the workdays and the leisure day. Regarding social isolation, we found a significant positive relationship with the Cortisol Awakening Response (CAR) on the leisure day but no significant relationship between social isolation and the CAR on the workdays or the diurnal cortisol slope (DCS). Additionally, we found no associations between social isolation and α-amylase measures. Lastly, our results indicated no significant correlation between social isolation and neuroticism after controlling for confounders and no significant interaction of neuroticism on the relationship between social isolation and the salivary biomarkers.

For our first aim, we compared cortisol and α-amylase concentrations between the two averaged workdays and the leisure day. The CAR was significantly higher on workdays than on the leisure day, which aligns with previous studies [59–62]. The anticipation of the workday may act as a stressor associated with an increased CAR, while leisure days may contribute to the teachers’ stress reduction. This may imply that teachers who over-commit themselves and do not take a day of rest during the workweek may be at higher risk of developing consistently increased HPA axis activity in the mornings, potentially affecting one’s physical and mental health in the long term. A further explanation may be that the higher CAR provides the organism with more energy that is needed on workdays [63, 64]. The DCS also differed significantly between the workdays and the leisure day, with a steeper decrease on working days than on the leisure day. This effect was due to higher levels in morning samples of workdays and no significant differences in evening levels between working and leisure days, which matches previous studies [65, 66]. The finding of increased DCS levels mainly due to higher morning cortisol levels on the workdays may be related to an increase in anxiety and stress levels [61, 67]. Furthermore, this finding indicates that the teachers in our sample were able to de-stress in the evening, even on workdays.

There were no significant differences in α-amylase concentrations regarding the AAR or the diurnal α-amylase slope, between the workdays and the leisure day. This is consistent with the preliminary study by Wettstein et al. [62] examining how workdays and leisure days may affect α-amylase concentrations in eight healthy teachers. In a study involving university students, Skoluda et al. [68] also found that weekdays or leisure days did not predict AAR. However, they found a positive association between AAR and wake-up time, suggesting a relationship between early wake-up time and increased AAR. Further studies with larger samples are needed to better understand the role of α-amylase concentrations concerning potential psychological stress on workdays compared to leisure days.

Regarding our second aim, we found a significant positive relationship between social isolation and the CAR on the leisure day and a weak positive association with the CAR on workdays (*p* = .051). Although certain previous findings were not consistent with this [31, 69], social isolation has often been associated with an increased CAR as an indicator of HPA-axis activation [28, 30, 70, 71]. As social isolation increases the sensitivity to social stimuli and disrupts individual "social homeostasis" [72], it seems to contribute to increased levels of psychosocial stress associated with hyperactivity of the HPA axis [28, 30]. The magnitude of CAR has also been associated with the anticipation of the coming day [64], and our results suggest that socially isolated teachers experience higher HPA activation in the morning compared to teachers who are not socially isolated. One explanation for the stronger relationship between social isolation and CAR on the leisure day than on the workdays in our sample may be that socially isolated teachers feel particularly isolated on weekends/ leisure days. This notion is supported by a study by Okamura et al. [73], who found that CAR on weekends was higher in the high loneliness group than in the low loneliness group. Furthermore, some studies showed that increased loneliness the previous day predicted increased CAR the following day [28, 30]. One explanation for these results may be that an increase in CAR due to feelings of loneliness on the previous day provides additional energy helping the individual to cope with the following day’s demands [30]. However, given the interval of up to several months between measuring social isolation and collecting saliva samples in our study, an increased CAR may also indicate a persistent maladaptive stress response due to chronic social isolation. This interpretation would support the results of a study by Steptoe et al. [29], who found a positive correlation between CAR and chronic social isolation.

Contrary to these findings, social isolation was not associated with DCS in our sample. In the literature, there is also conflicting evidence on whether social isolation affects the course of diurnal cortisol concentrations in adults. For example, whereas Grant et al. [70] and Lai et al. [31] found an association between social isolation and a steeper DCS, Steptoe et al. [29] found no association, and Doane and Adam [30] even pointed to an association with a flattened DCS. This inconsistency may be due to several methodological differences concerning sampling time, the number of measurements, the measurement of social isolation, the participant’s age, or a combination of these factors [18, 31]. The positive association between social isolation and CAR on the leisure day and null findings with DCS in our sample suggest that distinguishing between CAR and DCS may be particularly important when examining psychophysiological factors.

Whereas our results show an association between social isolation and salivary cortisol, there was no association between social isolation and salivary α-amylase measures. Previous studies simultaneously measuring salivary cortisol and α-amylase in a psychosocial context also reported inconsistent results. Our results align with a study by Bauduin et al. [33], who found no significant association between α-amylase and social withdrawal but a positive association between social withdrawal and salivary cortisol. In contrast, Ponzi et al. [34] showed lower pre-interview salivary cortisol concentration and α-amylase activity in children who reported high friendship densities. Van Veen et al. [35] found increased α-amylase but not cortisol in a clinical sample with social-anxiety patients. One explanation for these heterogeneous findings is that α-amylase may reflect the activity of a different stress system than the HPA axis, thus responding differently to stressors. Stressors with varying effects on the autonomic nervous system may have different outcomes regarding salivary variables [74]. In the literature on acute stress, salivary α-amylase is often used to measure activation of the sympathetic nervous system, though it is debatable whether or to which extent it also indicates parasympathetic nervous system activity. The different methodological details may also play a role in these somewhat contradictory results, as these studies differ in terms of clinical vs. non-clinical samples, sample sizes, acute vs. global measurements throughout the day, and natural vs. laboratory settings. In contrast to our study, none of the studies discussed above were conducted in everyday-life settings with eight measurement points to reveal accurate response patterns of α-amylase over three days. It appears that salivary α-amylase is a promising measure for short-term reactivity in non-clinical samples [15–17, 34, 75], but a less suitable indicator for global measurements throughout the day [33, 62].

The third aim of the present study was to investigate the moderating role of neuroticism on the relationship between social isolation and the salivary biomarkers. No significant moderations were found. Taken together, of all the examined cortisol and α-amylase measures, only the CAR on the leisure day correlated with social isolation. This association was found regardless of whether the teachers reported high or low neuroticism scores. Longitudinal studies with larger samples are still needed to clarify the moderating role of neuroticism on the relationships between social isolation and salivary biomarkers in teachers’ natural settings.

This study holds several limitations. First, the sample of the present study is relatively small, limiting the results’ generalizability. Second, different salivary glands may have different salivary secretion rates, influencing the amounts of sAA delivered into the oral fluid. In this study, our participants gave stimulated (chewing on cotton rolls), not non-stimulated (passive drooling) saliva samples, potentially altering sAA results [76, 77]. Third, the ecological validity of saliva sampling lacked an objective control of compliance (e.g., memotrack) and relied solely on our participants’ accuracy in following the sampling instructions and schedule. Although our low non-responder rate (results not presented) indicates high adherence to the sampling protocol and a healthy study population, we cannot exclude the possibility that our results were influenced by non-adherence to the sampling protocol. Ideally, future studies will objectively assess the time of sampling by using an electronic monitoring device such as memotrack and, in addition, an ECG and/or motility recording during the night and morning hours. Fourth, the assessment of the psychometric variables can be criticized. Given that our study was exploratory in nature, we did not use the full 12-item scale of the NEO-FFI [48] and no questionnaire on social isolation that precisely distinguished between subjective and objective social isolation. A replication of the present study might benefit from examining both subjective and objective social isolation to determine whether there are independent and differential effects on HPA and ANS activity. Fifth, a limitation is the varying time delay between psychometric assessment and saliva collection. However, when comparing correlations with and without control for time delay, we found no major change neither in the direction nor in the strength (or significance) of the associations. Similarly, the moderations and their interaction effects remain unaffected as well (results not presented). Sixth, seasonal variations could have affected salivary biomarkers. Conducted Kruskal-Wallis-test, however, showed no significant (asymptotic) seasonal effect of the salivary biomarkers (results not presented). Finally, it is important to note that the salivary measures were assessed before and during the pandemic. Although teachers were teaching face-to-face in the classroom as usual during the data collection period, the results should be interpreted accordingly.

Despite these limitations, this study has some strengths. First, we combined self-reported social isolation and neuroticism with biomarkers, as these traits might contribute to the individual stress experience. Furthermore, in contrast to the widely studied role of cortisol in stress research, α-amylase is a relatively new marker of ANS function and reactivity in the psychosocial context. Moreover, not only the awakening responses of cortisol and α-amylase but also the diurnal slopes throughout the day were assessed. This could be important as these measures may also indicate ANS and HPA axis dysregulation. Finally, the ambulatory-assessment design of the study on two workdays and one leisure day allows for a better understanding of these physiological processes in the teachers’ natural settings.

In view of the potentially severe mental and physical consequences of unaddressed social isolation [5, 30, 78, 79], developing and promoting interventions (e.g., cognitive behavioral therapy or self-compassion training [80]) to alleviate feelings of social isolation and to help diminish psychobiological responses to social threat appraisal is of paramount importance. For teachers, such interventions may help mitigate stress by reducing perceptual and cognitive biases that promote hypervigilance to negative information in their social environment [5]. Future studies should consider a broader distribution of teachers with regard to stress perception, i.e., by conducting an initial screening of stress and comparing biopsychosocial variables in teachers with low and high stress levels.

Taken together, our findings suggest differences in salivary cortisol concentrations between workdays and leisure days in teachers and a positive association between social isolation and morning cortisol concentrations mainly on the leisure day. However, no relationships were found with α-amylase, indicating that α-amylase is unlikely to be an essential biomarker of social isolation. Our findings provide additional evidence for the specificity of pathways linking psychosocial stress due to social isolation and HPA axis functioning. Finally, this study adds to the literature on the inconsistent relationship between neuroticism and salivary biomarkers and warrants further investigation in a larger study.

## Supporting information

S1 Data (XLSX)
